# The Different Association between Serum Ferritin and Mortality in Hemodialysis and Peritoneal Dialysis Patients Using Japanese Nationwide Dialysis Registry

**DOI:** 10.1371/journal.pone.0143430

**Published:** 2015-11-23

**Authors:** Yukio Maruyama, Keitaro Yokoyama, Takashi Yokoo, Takashi Shigematsu, Kunitoshi Iseki, Yoshiharu Tsubakihara

**Affiliations:** 1 Division of Nephrology and Hypertension, Jikei University School of Medicine, Tokyo, Japan; 2 Committee of Renal Data Registry, Japanese Society for Dialysis Therapy, Tokyo, Japan; Robert Bosch Hospital, GERMANY

## Abstract

**Background/Aims:**

Monitoring of serum ferritin levels is widely recommended in the management of anemia among patients on dialysis. However, associations between serum ferritin and mortality are unclear and there have been no investigations among patients undergoing peritoneal dialysis (PD).

**Methods:**

Baseline data of 191,902 patients on dialysis (age, 65 ± 13 years; male, 61.1%; median dialysis duration, 62 months) were extracted from a nationwide dialysis registry in Japan at the end of 2007. Outcomes, such as one-year mortality, were then evaluated using the registry at the end of 2008.

**Results:**

Within one year, a total of 15,284 (8.0%) patients had died, including 6,210 (3.2%) cardiovascular and 2,707 (1.4%) infection-related causes. Higher baseline serum ferritin levels were associated with higher mortality rates among patients undergoing hemodialysis (HD). In contrast, there were no clear associations between serum ferritin levels and mortality among PD patients. Multivariate Cox regression analysis of HD patients showed that those in the highest serum ferritin decile group had higher rates of all-cause and cardiovascular mortality than those in the lowest decile group (hazard ratio [HR], 1.54; 95% confidence interval [CI], 1.31–1.81 and HR, 1.44; 95% CI, 1.13–1.84, respectively), whereas associations with infection-related mortality became non-significant (HR, 1.14; 95% CI, 0.79–1.65).

**Conclusions:**

Using Japanese nationwide dialysis registry, higher serum ferritin values were associated with mortality not in PD patients but in HD patients.

## Introduction

Anemia is prevalent in patients with chronic kidney disease (CKD) and is associated with excess mortality and morbidity [1]. Since an erythropoietin deficiency is the chief contributing factor, erythropoietin-stimulating agents (ESA) have allowed major advances in the management of anemia among patients with CKD. Because of increased iron demand, ESA administration could induce an absolute iron deficiency that presents as low blood iron content together with low iron stores. Iron administration is indicated under these conditions, and Japanese anemia guidelines recommend administering iron to patients with transferrin saturation (TSAT) < 20% and < 100 ng/mL of serum ferritin [2], which is the most common marker of iron stores.

On the other hand, iron utilization is often defective in patients with CKD, resulting in a functional iron deficiency characterized by increased serum ferritin values. Serum ferritin is both an iron storage protein and an acute phase reactant. Systemic inflammation decreases intestinal iron absorption and inhibits the release of iron from stores and macrophages via hepcidin modulation, and hepcidin levels reflect both inflammatory status and anemia management in patients with CKD [3]. We previously reported that the active form of hepcidin, hepcidin-25, is associated with serum ferritin, TSAT, hemoglobin, C-related protein (CRP), sex, as well as treatment with ESA and iron in patients with CKD [4]. The association between serum ferritin and serum hepcidin-25 was particularly robust and positive.

Several observational studies have associated higher serum ferritin with higher rates of mortality, including infection-related mortality, in patients undergoing hemodialysis (HD) [5–7]. In contrast, two studies have associated lower serum ferritin with worse outcomes [8, 9]. Several guidelines recommend to use serum ferritin for the assessment of iron status and subsequent iron therapy [2, 10–12]. In these guidelines, however, the information for the patients undergoing peritoneal dialysis (PD) were insufficient because the effects of serum ferritin on mortality among PD patients have not been investigated.

The present study aimed to examine whether serum ferritin and other markers of anemia are associated with mortality in both HD and PD patients using a large cohort from the Japanese nationwide dialysis registry.

## Materials and Methods

The Japanese Society for Dialysis Therapy has conducted annual surveys of dialysis facilities throughout Japan. The surveys address epidemiological background, treatment conditions and the outcomes of treatment with dialysis. At the end of 2007, 275,242 patients were undergoing dialysis in Japan [13]. Data were obtained from the standard analysis file, JRDR-13101 with the permission of the Committee of the Renal Data Registry of the Japanese Society for Dialysis Therapy (JRDR). The study protocol was approved by the Medicine Ethics Committee of the Japanese Society for Dialysis Therapy. The study proceeded in accordance with the Declaration of Helsinki. Baseline data of 191,902 patients (age, 65 ± 13 year; male, 61.1%; median dialysis duration, 62 months), who had available clinical data including laboratory data and data on 1-year outcome were extracted. Among them, 172,672 (90.0%) underwent HD, 13,976 (7.3%) underwent hemodiafiltration (HDF), 3,734 (1.9%) underwent PD. Among HD patients, 162,818 (94.3%) underwent three sessions weekly.

Biochemical parameters including hemoglobin (Hb), serum iron, serum ferritin, total iron binding capacity (TIBC), serum albumin, creatinine (Cr), blood urea nitrogen (BUN), and CRP were measured using standard laboratory techniques at each center and TSAT was calculated as serum iron divided by TIBC.

Information about all-cause, cardiovascular and infection-related death were extracted from the data at the end of 2008. Cardiovascular death was defined as death caused by heart failure, pulmonary edema, acute myocardial infarction, arrhythmia, endocarditis, valvular disease, subarachnoid hemorrhage, cerebral hemorrhage, cerebral infarction and sudden death. Infection-related death was defined as death caused by sepsis, pneumonia, peritonitis, tuberculosis, HIV, influenza and other types of infection.

### Statistical analysis

Data are presented as means ± SD or medians and interquartile range (IQR). Values with P < 0.05 were considered significant. Patients undergoing PD or three HD sessions weekly were compared using Student’s *t*-test, the Wilcoxon rank sum test or the chi-square test. Spearman correlation coefficient (rho) was used to determine the relationships between clinical parameters. The hazard ratios (HR) and 95% confidence interval (CI) for all-cause, cardiovascular and infection-related mortality rates were assessed using Cox regression analysis with the confounding factors of age, sex, dialysis duration, underlying disease, comorbid disease and laboratory data, i.e. all parameters listed in Table 1. Data were statistically analyzed using JMP, version 10.0.2 for Windows (SAS Institute Inc., Cary, NC, USA) and STATA version 11.1 (STATA Corporation, College Station, TX, USA).

**Table 1:** Baseline characteristics of patients undergoing dialysis.

Variable		Whole Group	HD (thrice weekly)	PD	*P*
Number (%)		191902	162818 (84.8%)	3734 (1.9%)	
Age (years)		65±13	65±13	60±15	<0.01
Male (%)		117233 (61.1%)	99991 (61.4%)	2292 (61.4%)	0.97
Dialysis duration (months)		62 (26–123)	61 (27–119)	36 (16–68)	<0.01
Height (cm)		159±10	159±10	159±12	0.02
Body weight (kg)		56.0±11.9	56.1±11.9	59.2±12.4	<0.01
Underlying disease					<0.01
	Chronic glomerulonephritis (%)	79192 (41.3%)	65679 (40.3%)	1796 (48.1%)	
	Diabetic nephropathy (%)	63862 (33.3%)	56184 (34.5%)	933 (25.0%)	
	Nephrosclerosis (%)	12529 (6.5%)	10607 (6.5%)	324 (8.7%)	
	Polycystic kidney disease (%)	6645 (3.5%)	5566 (3.4%)	96 (2.6%)	
	Others or unknown (%)	29674 (15.5%)	24782 (15.2%)	585 (15.7%)	
Comorbidity					
	Acute myocardial infarction	10759 (6.4%)	9136 (6.4%)	190 (5.1%)	0.11
	Cerebral hemorrhage	6671 (4.0%)	5864 (4.1%)	101 (3.0%)	<0.01
	Cerebral infarction	19249 (11.7%)	16710 (12.0%)	260 (7.9%)	<0.01
	Quadruple amputation	4225 (2.5%)	3687 (2.6%)	47 (1.4%)	<0.01
Laboratory data					
	Hemoglobin (g/dL)	10.3±1.3	10.3±1.3	10.1±1.5	<0.01
	Iron (μg/dL)	63±29	63±29	79±34	<0.01
	Ferritin (ng/mL)	132 (52–270)	133 (53–273)	134 (68–240)	0.22
	TIBC (μg/dL)	237±61	236±61	248±63	<0.01
	TSAT (%)	25.6 (18.7–34.1)	25.6 (18.8–34.1)	30.6 (21.8–39.7)	<0.01
	Albumin (g/dL)	3.7±0.4	3.7±0.4	3.4±0.5	<0.01
	BUN (mg/dL)	66±17	67±17	54±16	<0.01
	Creatinine (mg/dL)	10.4±3.0	10.4±3.0	10.1±3.7	<0.01
	CRP (mg/dL)	0.12 (0.06–0.4)	0.12 (0.06–0.4)	0.17 (0.06–0.51)	<0.01

Means±SD or medians and interquartile range (IQR)

Abbreviations: HD, hemodialysis; PD, peritoneal dialysis; TIBC, total iron binding capacity; TSAT, transferrin saturation; BUN, blood urea nitrogen; CRP, C-reactive protein; PTH, parathyroid hormone.

## Results

Table 1 shows the baseline characteristics of the 191,902 patients (mean age, 65 ± 13 year; male, 61.1%; median dialysis duration, 62 months) included in the present study. Among them, 162,818 (84.8%) underwent three HD sessions per week and 3,734 (1.9%) received PD. The underlying diseases comprised chronic glomerulonephritis (CGN) in 79,192 (41.3%), diabetic nephropathy in 63,862 (33.3%), nephrosclerosis in 12,529 (6.5%), polycystic kidney disease (PKD) in 6,645 (3.5%), and others or unknown in 29,674 (15.5%). The numbers of patients with a history of acute myocardial infarction, cerebral hemorrhage, cerebral infarction, and quadruple amputation were 6.4%, 4.0%, 11.7% and 2.5%, respectively. Hb, serum iron, TSAT, and serum ferritin values were 10.3 ± 1.3 g/dL, 63 ± 29 μg/dL, 25.6% (18.7%–34.1%) and 132 (52–270) ng/mL, respectively. The HD patients were older, had a longer dialysis duration and more comorbidities, including cerebral hemorrhage, cerebral infarction and quadruple amputation. Diabetic nephropathy and PKD were the more prevalent underlying diseases in HD, whereas CGN and nephrosclerosis were more prevalent in PD patients. Values for Hb, BUN, Cr, and albumin were higher in HD patients, and those for CRP and TSAT were higher in PD patients. Serum ferritin did not significantly differ between the HD and PD groups (133 (53–273) vs. 134 (68–240); P = 0.22). Both in the HD and PD groups, serum ferritin was positively associated with CRP (rho = 0.11; P<0.01, rho = 0.14; P<0.01, respectively) and negatively associated with serum albumin (rho = -0.06; P<0.01, rho = -0.06; P<0.01, respectively).

**Fig 1:**
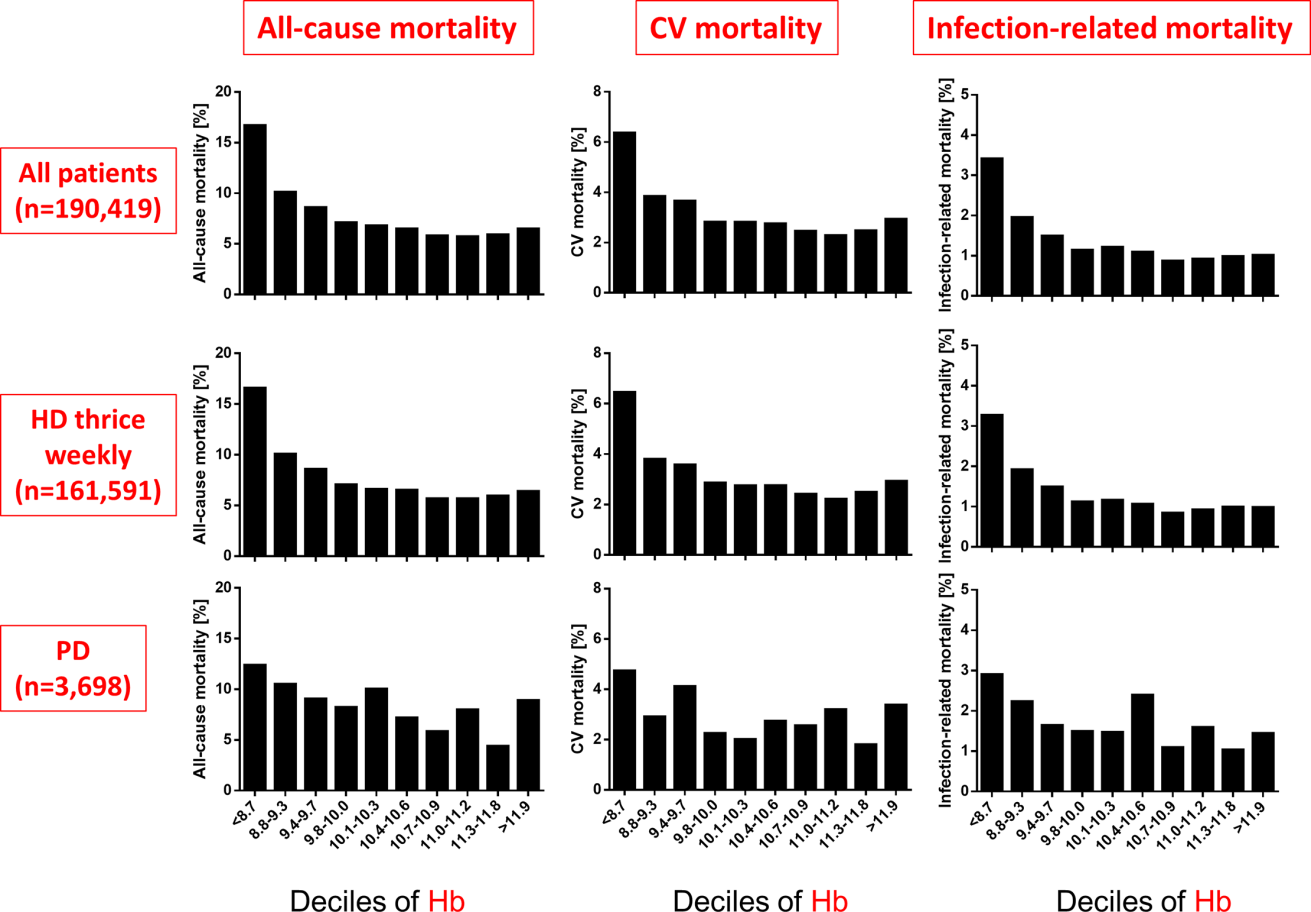
Comparison of all-cause, cardiovascular and infection-related mortality rates of deciles of hemoglobin levels. Cut-off levels: 8.8, 9.4, 9.8, 10.1, 10.4, 10.7, 11.0, 11.3, and 11.9 g/dL. CV, cardiovascular.

**Fig 2:**
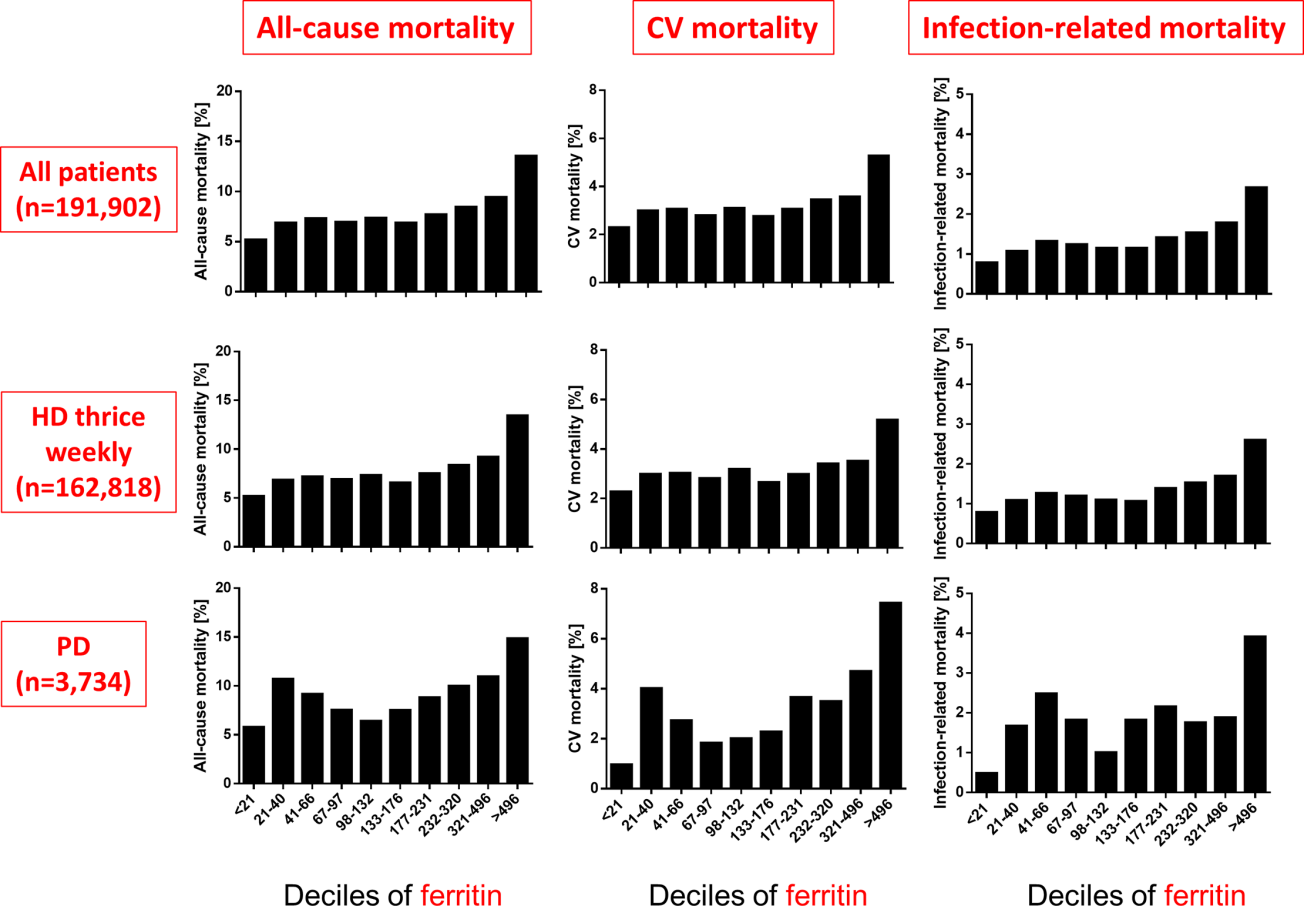
Comparing all-cause, cardiovascular and infection-related mortality of deciles of serum ferritin. Cut-off levels: 21, 41, 67, 98, 133, 177, 232, 321, and 496 ng/mL. CV, cardiovascular.

**Fig 3:**
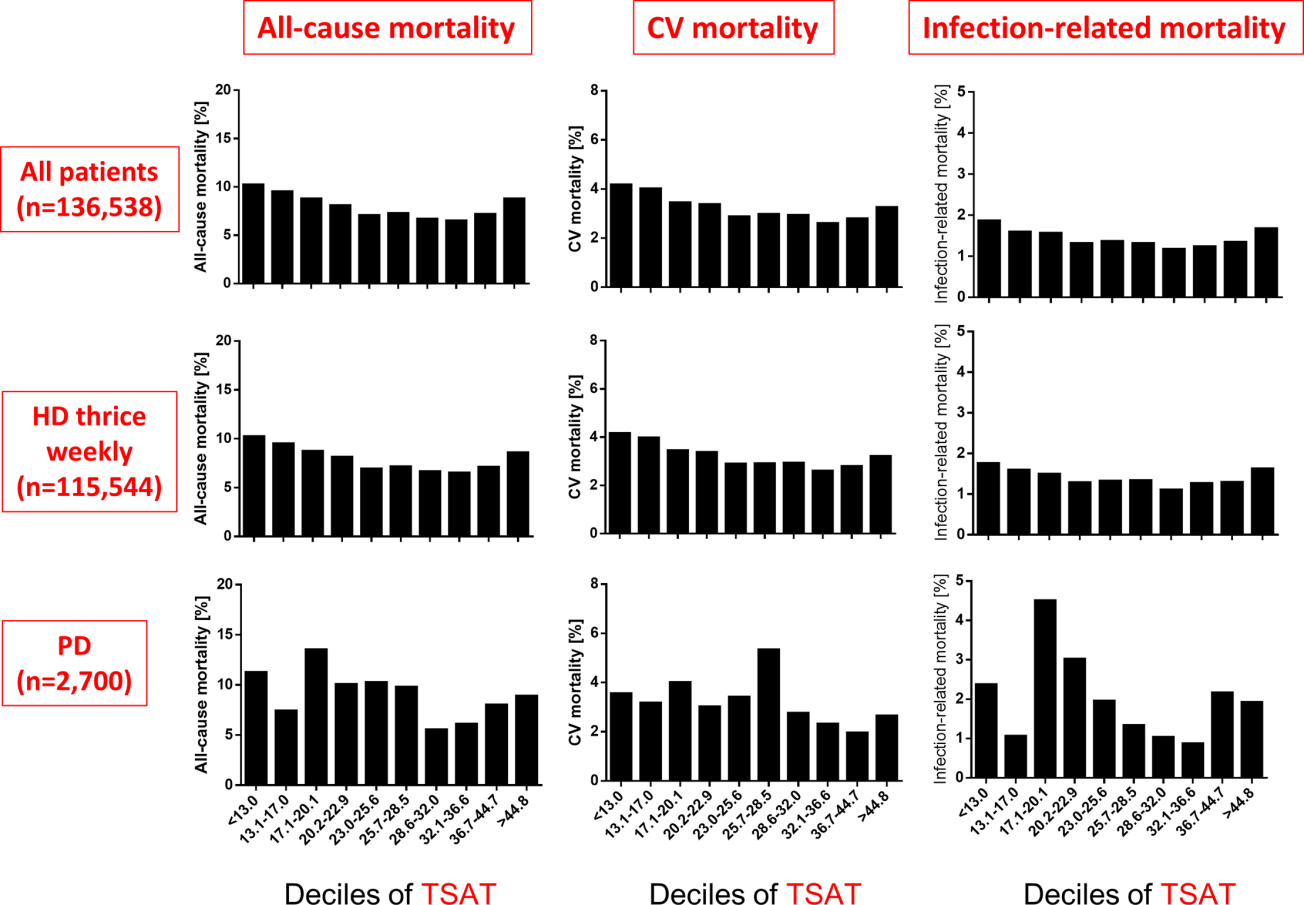
Comparison of all-cause, cardiovascular and infection-related mortality of deciles of transferrin saturation. Cut-off levels: 13.1%, 17.1%, 20.2%, 23.0%, 25.7%, 28.6%, 32.1%, 36.7% and 44.8%. CV, cardiovascular; TSAT, transferrin saturation.

Of the 191,902 patients, 15,284 (8.0%) died within one year, including 6,210 (3.2%) of cardiovascular causes and 2,707 (1.4%) infection-related causes. According to the type of dialysis, 12,800 (7.9%) died within one year, including 5,216 (3.2%) of cardiovascular causes and 2,230 (1.4%) infection-related causes in HD patients, and 333 (8.9%) died within one year, including 118 (3.2%) of cardiovascular causes and 70 (1.9%) infection-related causes in PD patients. Lower Hb levels among HD patients were associated with increased all-cause, cardiovascular, and infection-related mortality rates (Fig 1). Although higher serum ferritin was associated with higher all-cause, cardiovascular, and infection-related mortality rates, these associations were not linear and remarkable especially among groups with > 496 and <21 ng/mL (Fig 2). The association between TSAT and mortality was U-shaped (Fig 3). Associations between mortality and these three parameters were weak among PD patients (Figs 1–3).

In HD patients, higher serum ferritin was associated with all-cause, cardiovascular, and infection-related mortality in univariate analysis (HR, 1.0040; 95% CI, 1.0038–1.0043; HR, 1.0035; 95% CI, 1.0031–1.0039 and HR, 1.0045; 95% CI, 1.0040–1.0049, per 10-ng/mL respectively), and these associations were attenuated but remained significant after adjustment for age, sex, dialysis duration, underlying disease, comorbid disease and laboratory data (HR, 1.0018; 95% CI, 1.0012–1.0023; HR, 1.0016; 95% CI, 1.0006–1.0025 and HR, 1.0016; 95% CI, 1.0003–1.0028, per 10-ng/mL respectively). In PD patients, higher serum ferritin was also associated with all-cause, cardiovascular, and infection-related mortality in univariate analysis (HR, 1.0070; 95% CI, 1.0045–1.0090; HR, 1.0086; 95% CI, 1.0055–1.0108 and HR, 1.0064; 95% CI, 1.0003–1.0103, per 10-ng/mL respectively), whereas these associations became non-significant in multivariate analysis (HR, 1.0003; 95% CI, 0.9896–1.0095; HR, 1.0036; 95% CI, 0.9898–1.0151 and HR, 0.9801; 95% CI, 0.9409–1.0073, per 10-ng/mL respectively).

**Fig 4:**
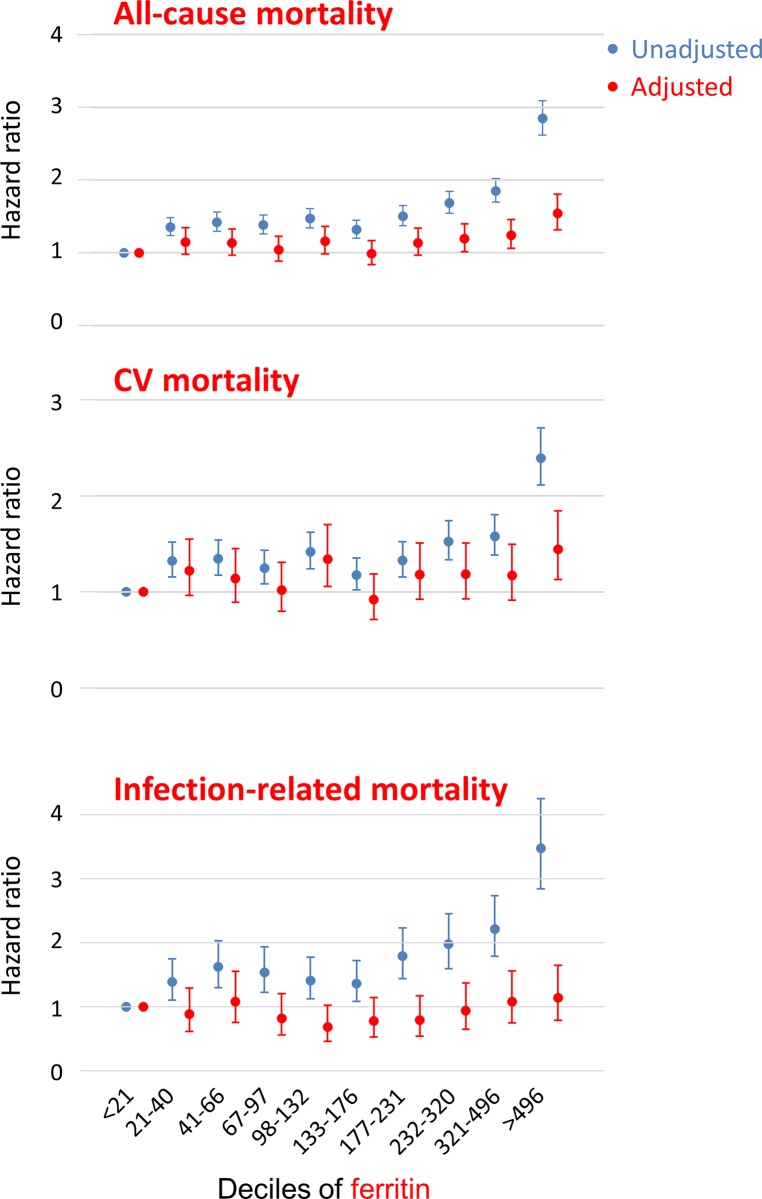
Hazard ratios for all-cause, cardiovascular and infection-related mortality by deciles of serum ferritin. Values are shown as hazard ratios and 95% confidence intervals. Adjusted for age, sex, dialysis duration, height, body weight, underlying disease, comorbidity, hemoglobin, iron, transferrin saturation, albumin, blood urea nitrogen, creatinine and CRP. CV, cardiovascular.

To examine the associations of serum ferritin with mortality in detail, we performed Cox regression analysis using serum ferritin as decile variables in HD patients (Fig 4). Univariate analysis showed that patients in the highest group of serum ferritin had higher rates of all-cause, cardiovascular, and infection-related mortality than those in the lowest group (HR, 2.84; 95% CI, 2.62–3.09; HR, 2.39; 95% CI, 2.11–2.71 and HR, 3.48; 95% CI, 2.84–4.25, respectively). After adjustment for confounders, the associations between serum ferritin and all-cause and cardiovascular mortality rates were attenuated but remained significant (HR, 1.54; 95% CI, 1.31–1.81 and HR, 1.44; 95% CI, 1.13–1.84, respectively), whereas that with infection-related mortality became non-significant (HR, 1.14; 95% CI, 0.79–1.65).

## Discussion

The major finding of this cohort study of 191,902 dialysis patients was that higher serum ferritin values were significantly associated with not only all-cause, but also cardiovascular mortality rates. These associations were not linear and remarkable especially among groups with > 496 and <21 ng/mL of serum ferritin. These positive associations between higher serum ferritin values and higher mortality rates are consistent with previous findings of HD patients [5–7]. Kalantar-Zadeh et al. [5] found no significant differences in risk for all-cause and cardiovascular mortality among 58,058 HD patients with serum ferritin levels of 200 to 1,200 ng/mL, whereas those with serum ferritin levels of ≥ 1,200 ng/mL were significantly associated with increased mortality rates. Japanese HD patients notably have very low mortality and morbidity rates, are treated with low doses of ESA and have low Hb levels according to the Dialysis Outcomes and Practice Patterns Study (DOPPS) [14–16]. Additionally, Japanese patients typically receive lower doses of intravenous iron and have considerably lower ferritin levels than patients in Western countries, although TSAT levels are similar [16]. Kuragano et al. [6] found in a prospective, observational, multicenter study of 1,086 Japanese HD patients that hyperferritinemia, defined as serum ferritin > 100 ng/mL, is a risk factor for cardiovascular disease, infection, hospitalization and death. Here, we divided serum ferritin values into finer categories, not by specific cut-off value, and found that serum ferritin values of < 21 and > 496 ng/mL, were particularly associated with mortality among Japanese HD patients.

In the present study, serum ferritin was associated with infection-related mortality in univariate analysis, but this association became non-significant in multivariate analysis. Ferrokinetics and infection are closely interlinked in CKD patients. Intravenous iron promotes apoptosis and inhibits phagocytic activities of polymorphonuclear leukocytes (PMN) [17]. A systematic review found that although intravenous iron supplementation helps to increase hemoglobin and reduce the likelihood of requiring red blood cell transfusions, it is also associated with a significant increase in risk of infection compared with oral or no iron supplementation [18]. Although the findings have been contradictory, several studies have established associations between higher serum ferritin values and infection complications [19]. Galic et al. [20] reported that the incidence of sepsis and vascular access infection is higher among HD patients who have serum ferritin values > 500 ng/mL. The lack of association between serum ferritin and infection-related mortality in the multivariate analysis including CRP, albumin and several comorbidities indicated that serum ferritin acts not only as iron storage but as acute phase reactant. Indeed, CKD patients with hyperferritinemia are associated with several conditions including inflammation, infection, liver disease and malignancies as well as iron overload [21].

We also found that higher Hb values were also associated with a modest but significant increase in mortality rates among HD patients. Akizawa et al. [22] found that mortality rates among a population of 10,310 individuals with incident HD tended to be higher among those with Hb levels of > 12, than 10–11 and 11–12 g/dL. Several randomized controlled trials have also shown that targeting Hb levels of 13 g/dL increases mortality or morbidity rates, and that clinicians need to be aware of these effects of higher Hb levels [23–25].

The present study included 3,734 PD patients, which accounted for one-third of all PD patients in Japan, but associations between anemia-related parameters and mortalities were unclear. The associations between Hb, serum ferritin and TSAT and mortality were weak, and multivariate Cox regression analysis revealed that serum ferritin was not independent determinant of mortality in this population. Little is known about associations between anemia management and the outcomes of PD patients compared with HD. Li et al. [26] reported that Hb levels of < 11 g/dL were associated with higher mortality rates than those of 11–11.9 g/dL using 13,974 patients on incident PD. Similarly, Molnar et al. [27] reported that Hb levels of < 11 g/dL were associated with higher mortality rates than those of 12–13 g/dL among 9,269 ESA-treated PD patients. To the best of our knowledge, associations between serum ferritin and mortality have not been studied in PD patients. There are major differences in the management of anemia between HD and PD patients. Wetmore et al. [28] compared the anemia management of these groups using a large cohort of more than two hundred thousand dialyzed patients, and found that use of both ESA and intravenous (iv) iron was lower in PD despite of the lower hemoglobin in this group. Commonly, PD patients have fewer monthly interactions with their health care providers, and anemia treatments, including ESA and IV iron are limited. In the present study, PD patients had higher CRP and thought to be more inflamed. Since serum ferritin is both an iron storage protein and an acute phase reactant, similar serum ferritin between HD and PD might indicate both lower doses of IV iron and inflammatory condition. However, we have to say that the characteristic of Japanese PD patients, including younger age, shorter dialysis duration and probably better adherence to the therapies, make the comparisons of clinical biomarkers and outcome difficult. Since the clinical presentation of anemia widely varies between patients on HD and those on PD due to factors such as the preservation of residual renal function, blood loss during HD sessions, different profiles of uremic toxin removal, ESA dose and iron administration, further study is needed to establish targets for anemia management among PD patients [28–30]. Although definite reason of the lack of association between serum ferritin and mortality in PD patients is not clear from the results of the present study, we speculate that variation in several parameters such as dose of dialysis, residual renal function, ESA and iron administration and inflammation could affect mortality, and these effects overcame the impact of serum ferritin.

The present study has several limitations. First, we did not have a control group, and the observational design allowed only limited conclusions. Especially, we cannot prove cause-and-effect relationship. Second, serum ferritin values and other laboratory data were measured only at baseline, therefore we could not determine the effect of changes from baseline during follow-up using time-dependent analyses. Additionally, follow-up period of 1 year was short. Third, information about ESA and iron administration were not collected, thus complicating any interpretations regarding the effects of serum levels of ferritin, TSAT and Hb. Additionally, comparisons of mortality between HD and PD patients were difficult because the dose and mode of the administration of these drugs were different between these two groups.

In summary, serum ferritin is associated with not only all-cause, but also cardiovascular mortality in Japanese HD patients. Serum ferritin values of <21 and > 496 ng/mL were particularly associated with lower and higher mortality rates, respectively. On the other hand, serum ferritin was not obviously associated with mortality among PD patients.
